# Development of Novel Whey-Mango Based Mixed Beverage: Effect of Storage on Physicochemical, Microbiological, and Sensory Analysis

**DOI:** 10.3390/foods12020237

**Published:** 2023-01-04

**Authors:** Tanvir Ahmed, Ashfak Ahmed Sabuz, Anirudha Mohaldar, H. M. Sazzad Fardows, Baskaran Stephen Inbaraj, Minaxi Sharma, Md Rahmatuzzaman Rana, Kandi Sridhar

**Affiliations:** 1 Department of Food Engineering and Tea Technology, Shahjalal University of Science and Technology, Sylhet 3100, Bangladesh; 2 Postharvest Technology Division, Bangladesh Agricultural Research Institute (BARI), Gazipur 1701, Bangladesh; 3 Department of Food Science, Fu Jen Catholic University, New Taipei City 242 05, Taiwan; 4 Haute Ecole Provinciale de Hainaut-Condorcet, 7800 Ath, Belgium; 5 Department of Food Technology, Koneru Lakshmaiah Education Foundation Deemed to be University, Vaddeswaram 522 502, Andhra Pradesh, India

**Keywords:** whey, mango, physico-chemical properties, functional beverage, shelf-life

## Abstract

The present study was aimed at developing whey-mango-based mixed beverages and characterizing their physicochemical properties. Three different formulations were prepared by varying proportions of whey and mango (sample-1 = 60:20 mL, sample-2 = 65:15 mL, and sample-3 = 70:10 mL). Prepared beverage samples during 25 days of storage revealed a significant increase in acidity (0.27 ± 0.02–0.64 ± 0.03%), TSS (17.15 ± 0.01–18.20 ± 0.01 °Brix); reducing sugars (3.01 ± 0.01–3.67 ± 0.01%); moisture (74.50 ± 0.02–87.02 ± 0.03%); protein (5.67 ± 0.02–7.58 ± 0.01%); fat (0.97 ± 0.01–1.39 ± 0.04%); and carbohydrate (18.01 ± 0.02–3.45 ± 0.02%). The sedimentation rate was only 1%. The total plate count for the prepared samples ranged from 3.32 ± 0.08 to 3.49 ± 0.15 log CFU/mL while yeast and mold counts varied between 0.48 ± 0.01 to 1.85 ± 0.11 Log CFU/mL. The coliform count was below the detection limit (<1). The overall sensory score revealed that the whey beverage with more mango juice could attain acceptable quality upon processing. Based on the findings, it may be concluded that whey can be utilized with fruits and vegetables to develop whey-based beverages.

## 1. Introduction

The food industry is currently looking for ingredients which might provide good functional and nutritional characteristics to manufacture different food items with value added. The increased knowledge of consumers about nutrition, health, and food quality, as well as the high level of competition in the market is forcing the food industry to search for these ingredients. In this context, a group of novel functional food products has been developed throughout recent years. Among these, over 40% of functional foods are dairy products and fermented beverages containing milk whey are the principal functional beverages [1]. Whey is a valuable by-product liquid obtained from protein precipitation in milk from the cheese industry [2]. Moreover, over the years, several methods have been explored to convert huge volumes of whey into food-grade products. The reason behind this is that only about 60% of whey generated globally is used to manufacture products. The remainder of the material is disposed of as waste [3]. Traditionally, whey has been discarded in surface water or fed to cattle. Whey dumping on soil causes serious environmental pollutants by changing the physico-chemical properties of soil, which leads to lower crop yields. Furthermore, whey waste can also decrease dissolved oxygen and inhibit biodegradability when it is released into bodies of water, and causes a serious threat to aquatic life and human health [4]. In addition, existing environmental restrictions and levies force cheese producers to treat whey before disposal, and the costs of transporting entire whey to feed uses have become challenging as a result of industrial centralization [5]. On the other hand, whey contains various nutrients, such as calcium, magnesium, phosphorus, vitamins (riboflavin and thiamine), and protein (α-lactoalbumin, β-lactoglobulin, immunoglobulins, serum albumin), which accounts for around 85–95% of the volume of milk, and it retains up to 55% of its contents after processing [6,7,8,9]. Considering the nutrient value and wastage of milk whey, food industries are trying to develop new combinations of food products based on whey. Given the importance of the growing global food crisis and environmental concern, returning whey into the human food chain in a palatable way would be a more logical and economical method than disposal.

Although numerous attempts have been made to incorporate whey into the composition of various dairy products, there is still much room to explore the possibility of its uses in the beverage industry. In recent years, numerous patents regarding the preparation of whey beverages with the addition of various ingredients have been registered. For example, a variety of citrus fruits, tropical fruits (mango, banana, grapefruit, papaya, tangerine, etc.), and other fruits such as berries, apples, cherries, pear, apricot, or melon have already been applied to develop whey-based beverages [10]. In addition to fruits and vegetables, a few researchers have also used oats, vanilla, cocoa, rice, chocolate, mint, and other suitable flavoring agents [11]. However, the main drawback in the case of all these recipes especially when whey is mixed with fruits like bananas, apples, and pears, is that sedimentation can occur due to a high volume of dry fruit matter and protein interactions with the components of dry fruit. The sediment accumulates over time, and hence these whey beverages do not perform well on the market [12]. On the other hand, the final product does not have good sensory attributes, such as color, flavor, and smell, if there is not enough dry fruit matter. In addition, certain fruits, due to their strong acidity, bitterness, and astringency, have poor taste and flavor and hence remain neglected [13].

Apart from these drawbacks, some people may be also allergic to whey protein, while others adhere to vegetarianism and maintain cholesterol-restricted diets [14]. On that account, the manufacturing of a beverage with a combination of fruits, vegetables, and whey can be a promising solution for enhancing the acceptance, nutrients, and sensory properties of whey-based beverages. Among the fruits and vegetables that have high production and industrial processing, mango and carrot stand out due to their low cost, high availability of nutrients, and palatable flavor. Mango and carrot are also enriched in functional components, such as phenolic compounds, ascorbic acid, fiber, vitamins, minerals, alpha- and beta-carotene, lutein, lycopene, etc. [15]. On the other hand, several researchers have recommended almond and green tea extract for their anti-allergic potential, and as a flavor-enhancing agent [16,17]. Almond contains a higher content of essential fatty acids (without cholesterol), vitamins, and minerals, while a few studies have demonstrated that green tea effects last longer with whey, and the combination of green tea extract and whey may have the potential, as a beverage, to fight muscle loss and burn more fat [17,18]. Furthermore, to increase the shelf-life of the beverage along with enhancing the flavor, aroma, and taste, some researchers have suggested including citric acid in the recipe because citric acid can lower the pH, control the growth of microorganisms, and improve beverage storage ability [19].

Many attempts to develop whey-based beverages by using only mango pulp or powder with different concentrations have been already reported. For example, Pandey and Ojha [20] prepared mango-based whey beverages with 599–749 mL whey and 150–350 mL mango pulp. In a study, Alane et al. [21] used 82–87 mL whey and 5–10 g mango pulp to formulate whey-based mango herbal beverages. In another study, Chavan et al. [22] developed a whey-based mango beverage using 77.24 g whey, 8.65 g mango pulp, and 0.19 g mango flavor. It is clear from previous studies that researchers are facing challenges in identifying optimal recipes for mixing fruit concentrates and/or other ingredients with fresh whey. There is scope to improve the quality of a whey-based beverage with acceptable sensory properties using mango combined with other ingredients. Thus, this study aimed to develop a whey-mango-based mixed beverage with varying concentrations of whey and mango along with a constant concentration of carrot, almond, green tea extract, sugar, and citric acid. The present study also evaluated the physico-chemical, microbiological, and sensory characteristics of prepared whey-based beverages soon after manufacture and during the storage period in the refrigerator (4 ± 1 °C) until 25 days.

## 2. Materials and Methods

### 2.1. Chemicals

All the chemicals used in this study were of analytical grade and obtained from Sigma-Aldrich (St. Louis, MI, USA) and Merck (Darmstadt, Germany).

### 2.2. Preparation of Milk Whey

Raw milk was purchased from the local market of Sylhet city, Bangladesh. Whey was prepared with few modifications following the Baljeet et al. [23] procedure. In brief, raw milk was heated to 82 °C with constant stirring and acidified by adding 2% citric acid at the rate of 2 g/kg of milk, which led to milk protein coagulation (casein). The coagulum was then filtered using a muslin cloth to drain and collect the generated whey. The pH of the obtained whey was adjusted to pH 5.0 using 10% NaHCO_3_ [24]. Afterward, the obtained whey was stored at 4 ± 1 °C in the refrigerator for further use.

### 2.3. Preparation of Mango Juice

Fresh mangoes (*Mangifera indica* L.) were purchased from the local market of Sylhet city, Bangladesh. The ripened mangoes were cleaned with hot water (55 °C). The selected mangoes were cut into small pieces with a sterile knife after removing the peel and seeds. The juice was extracted aseptically by using a juice extractor (model TRK-74, Turbora, Thailand). Then, the juice was refined by passing it through a muslin cloth and was kept under refrigerated condition (4 ± 1 °C) for further use.

### 2.4. Preparation of Carrot Juice

Mature carrots (*Daucus carota* subsp. *Sativus*) were collected from the local market of Sylhet city, Bangladesh for the experimental work. First, carrots were rinsed with hot water (55 °C) and then washed with distilled water. The carrots were hand-peeled with a knife and cut into small pieces. Afterwards, the juice was extracted from the carrots using a juice extractor (model: TRK-74, Turbora, Thailand) in an aseptic manner. The juice was strained with a muslin cloth to eliminate stone cells, prevent coarse particles, and include only fine particles with a colloidal coherence. The juice was stored at a refrigerated temperature (4 ± 1 °C) until used.

### 2.5. Preparation of Green Tea Extract

Green tea (*Camellia sinensis*) extract was prepared following the method described by Kawakastu et al. [25], with slight modifications: 10 g/50 mL of refined green tea (Department of Food Engineering and Tea Technology, SUST, Bangladesh) was steeped and extracted for 10–15 min. in 10 L of hot water at 80–100 °C with moderate agitation (1500 rpm). The extract was then filtered with a no. 4 Whatman filter paper. The extract was promptly cooled to a temperature of 25 °C, and then used as a green tea extract in the experiment.

### 2.6. Preparation of Almond Extract

Large quantities of almonds (*Prunus dulcis*) were obtained from the local market of Sylhet city, Bangladesh. The almond extract was prepared by slightly modifying the technique of Erdogan and Aygun [26]. First, the almonds were sorted and soaked in distilled water for 24 h. With the help of a knife, the peels were removed from the almonds, which were then crushed using a clean sterilized blender (model: Osterizer 857, Willamette Industries, Portland, Oregon, USA). The extract was then filtered using a muslin cloth to remove the chaff and then stored in refrigerated condition for further use.

### 2.7. Preparation of Whey-Mango-Based Mixed Beverage Modifications

Three different beverages were prepared with different proportions of whey and mango coded as sample-1(60 mL whey, 20 mL mango), sample-2 (65 mL whey, 15 mL mango), and sample-3 (70 mL whey, 10 mL mango). In each sample, 10 mL carrot, 6 mL sugar, 1.5 mL almond, and 1.5 mL green tea extract were used as constant (Table 1). A different formulation of beverages was made by heating whey to 50 °C with the addition of sugar. The mixture was then filtered through a muslin cloth after proper mixing. Subsequently, mango juice, carrot juice, green tea extract, almond extract, and 1% citric acid was added to the whey and homogenized. The produced beverages were then filtered and placed in previously sanitized glass bottles (250 mL), leaving 2.5 cm headspace, and sealed with a crown cork for airtightness. Later, the filled bottles were pasteurized at 85–90 °C for 10–15 min. and cooled at room temperature. The samples were then stored in a refrigerator at ambient temperature (4 ± 1 °C) for further study.

### 2.8. Storage Studies

The beverage samples were aseptically filled in PTE bottles. Beverage bottles were stored in a refrigerated condition (4 ± 1 °C) for 25 days. At 5-day intervals, samples were taken for analysis of their physico-chemical properties, microbiological analysis, and sensory evaluation.

#### 2.8.1. Physicochemical Analysis

##### pH Value

The pH measurement of beverage samples was performed three times using a digital pH meter (model: 111E, Electronics India, India). The pH meter was calibrated according to the manufacturer’s instructions using the commercial buffer solutions of pH 7 and pH 4 before measurement. After stabilization, a pH electrode was inserted into a sample of approximately 30 mL, and the pH was recorded.

##### Determination of Titratable Acidity

The titration method developed by Ranganna [27] was used to determine the titratable acidity of beverage samples. The acidity of each beverage sample was analyzed by titrating 10 mL of each beverage sample against the standard 0.1 N NaOH solution until the substance reached a pH value of 8.2 using phenolphthalein as an indicator, and the result was expressed in terms of lactic acid.

##### Total Soluble Solids (TSS) Determination

A digital refractometer (model: 300036; Sper Scientific, Scottsdale, AZ, USA) was used to determine the total soluble solids (TSS) contents at ambient temperature (25–30 °C). Before determining the TSS in the sample, the refractometer was calibrated with distilled water. The TSS value was determined by placing a drop of beverage sample on the refractometer’s prism. TSS was calculated using an average of five readings and reported as °Brix.

##### Determination of Moisture

Moisture contents were measured using the Association of Official Analytical Chemists (AOAC) [28] recommended oven-drying method. At first, the weights of three empty dry crucibles were taken and 10 mL of beverage samples were taken in each dried crucible. Then, the crucibles with samples were dried in an air oven (model: YCO-No 1, Yohmai, French) at 100 °C until the constant weight was obtained after three successive weight measurements (model: DLX-061, E G Kantawalla Pvt Ltd., Mumbai, Maharashtra, India). The crucibles were cooled in desiccators. Later, the crucibles were removed from desiccators and weighed soon after reaching room temperature.

##### Determination of Ash

According to the method described by the AOAC [28], at first, an empty crucible was accurately weighed. Then, 10 mL of samples were weighed in dry crucibles and carbonized on a hot plate before being heated for 5 h at 550 °C in a muffle furnace (model: FN 100, Nüve, Turkey). Later, it was cooled in a desiccator and weighed.

##### Determination of Fat

Fat content was determined using the AOAC- [28] designed Soxhlet extraction method. A thimble was cleaned, dried, and weighed, and then a 5 g oven-dried concentration of beverage sample was added and weighed again. The petroleum ether (40–60 °C) was added to a round bottom flask up to a third quarter of the flask. A reflux condenser was attached to a Soxhlet extractor (model: ASTM E 438, Fisher Scientific, Ottawa, ON, Canada) to adjust the heat source so that the solvent boiled gently. The sample was placed in the thimble, inserted into the Soxhlet, and the extraction with petroleum ether was performed for 6 h under reflux. Following that, the extractor’s barrel was emptied, and the condenser and thimble were removed and placed in an oven set to 100 °C for 1 h before being cooled in the desiccator and weighed again.

##### Determination of Protein Content

The percentage of the protein in the fortified whey beverage sample was determined by Pyne’s method [29]. Briefly, 10 mL of the homogenous sample was transferred into a 100 mL of volumetric flask. After that, 5 drops of phenolphthalein were added into the flask as an indicator. Then, 0.4 mL of saturated potassium oxalate was added to the sample and kept aside for 2–4 min. Later, the mixture solution was titrated against 0.1 N sodium hydroxide (standard alkali) to its endpoint. To this titrated solution, 2 mL of neutral formalin was added and mixed well. Subsequently, the mixture was titrated against the standard alkali to the same endpoint as before. The volume of the alkali used in the second titration was recorded.

##### Determination of Carbohydrate Content

Carbohydrate content was determined using a mathematical function described by the AOAC [28].

##### Determination of Ascorbic Acid Content

The ascorbic acid content in the beverage samples was determined according to the method described by Alam et al. [30]. In a 250 mL conical flask, 20 mL of sample solution was added first, followed by 150 mL distilled water and 1 mL of the starch indicator solution. Then, the sample was titrated with 0.005 mole L^−1^. The endpoint of the titration was determined by the development of starch–iodine complexes as the first noticeable trace of a dark blue-black color. Titrations were performed with additional aliquots of sample solution until concordant findings were obtained (titers agreeing within 0.1 mL).

##### Determination of Total Reducing Sugars

The reduced sugar content of the beverage samples was determined as described by Lane and Eynon [31]. A 20 mL of each beverage sample was added to 5 mL conc. HCL. After approximately 10–15 min. of heating, 5 mL of conc. NaOH solution was added to neutralize the solution and later diluted with 0.1 N NaOH solution. Furthermore, 5 mL of Fehling solution A and 5 mL of Fehling solution B were taken in another conical flask. Following that, the mixture in the flask was heated for 2–4 min. and then titrated with methylene blue as an indicator against the sample solution until the yellow-red ppt was attained. The endpoint for each sample was noted. Fehling solution A, Fehling solution B, and the factor of Fehling were prepared following the procedure described by Lane and Eynon [31].

##### Determination of Solid-Not-Fat (SNF) Content

A lactometer test was carried out to determine the solid-not-fat content of the beverage samples [32]. At first, the beverage sample was incorporated into a cylinder and warmed in a water bath (model: FSGPD28, Fisher Scientific, Ottawa, ON, Canada) maintained at 40–45 °C for 2–5 min. Then, the sample was cooled down to 29 °C. To avoid the production of foam, the sample was thoroughly mixed. In addition, the cylinder was filled with a sufficient amount of the sample to allow the free-floating of the lactometer. Afterwards, a Zeal lactometer (model: SKU: JG28101-8303, Japson, Tokyo, Japan) was placed in the sample and allowed to float in the sample until it stopped and assumed a constant level. The lactometer reading and the temperature of the sample were simultaneously recorded. A further reading was obtained by fluttering the top of the lactometer stem and assuming a constant level again. Then, the average of the two readings was considered and the solid-not-fat (SNF) content was calculated.

##### Determination of Mineral Content

Phosphorus (P) contents of the beverage samples were determined using the flame photometry method recommended by AOAC [33]. The calcium (Ca) and iron (Fe) content of the beverage samples were determined using an atomic absorption spectrophotometer (model: itachi Z6100, AAS, Tokyo, Japan) following wet digestion of the sample ash. All determinations were carried out in triplicates and the results were expressed as mg/100 mL.

##### Sedimentation

The sedimentation test was carried out in accordance with the approach reported by Gad et al. [34]. Whey-mango-based mixed beverage samples were centrifuged (model: Hettich Mikroliter D-7200, Diamed Lab Supplies Inc., Mississauga, ON, Canada) at 3000× *g* for 30 min. at ambient temperature. Sedimentation was expressed as a percentage of the total fluid weight.

#### 2.8.2. Microbiological Analysis

The standard method used by the American Association of Cereal Chemists (AACC) 42-11 [35] was employed for the estimation of total plate count using the pour plate method. The total plate count agar plates were incubated at 36 ± 1 °C for 48 h. The AACC 42–50 [35] method was used for yeast, and mold counts with the same dilutions to carry out the pour plate method. The plates were incubated for 3–5 days at 25 ± 1 °C. The coliform bacteria were enumerated on lauryl sulfate tryptone broth agar and incubated at 37 °C for 24–48 h [36]. The determinations were performed in triplicates and the counts were expressed as log CFU (colony forming units) per mL of solution.

#### 2.8.3. Sensory Evaluation

The sensory attributes for different treatment samples were evaluated following the 9-point hedonic scale rating. On this scale, ‘like extremely’ was given the highest score of ‘9’ and ‘dislike extremely’ is given the lowest score of ‘1’ [37]. Others were given intermediate scores according to the preferences of panel members. A judgment panel of 30 participants comprised of postgraduate students and faculty of the Food Engineering and Tea Technology, Shahjalal University of Science and Technology (SUST) was formed to evaluate various sensory parameters i.e., color, flavor, taste, appearance, and overall acceptability of the products.

### 2.9. Statistical Analysis

All tests were conducted in triplicate and data were analyzed by GraphPad Prism version 9 (GraphPad Software Inc., San Diego, CA, USA). Results are shown as mean values ± standard deviation. Statistical significance between the groups was evaluated by two-way analysis of variance (ANOVA) followed by Tukey’s multiple comparison test. A *p*-value of < 0.05 was considered statistically significant (5% probability).

## 3. Results and Discussion

The whey-mango-based mixed beverages were prepared in the laboratory of the Food Engineering and Tea Technology Department, Shahjalal University of Science and Technology, Sylhet-3100, Bangladesh for the present study. The whey and mango juice were the higher concentrations in the prepared whey-mango-based mixed beverages compared with other ingredients as shown in Table 1. In the present study, three formulations were designed for the preparation of whey-mango-based mixed beverages using different levels of whey (60–70 mL), mango (10–20 mL) with the constant concentration of carrot (10 mL), sugar (6 mL), almond (1.5 mL), green tea extract (1.5 mL), and citric acid (1 mL). The manufactured whey-mango-based mixed beverages were analyzed for sensory, physico-chemical, and microbiological properties throughout the storage period. The pH, acidity (%), TSS (°Brix), reducing sugar (%), ascorbic acid (mg/100 mL), SNF (%), moisture (%), protein (%), fat (%), carbohydrate (%), ash (%), minerals (calcium, phosphorus, and iron), and sedimentation were analyzed for physico-chemical properties. The microbiological analysis was carried out for the total plate count, yeast, and mold count as well as coliform count. The characteristics of various parameters are described below:

### 3.1. pH

The changes in pH of formulated whey-mango-based mixed beverages are illustrated in Table 2. The pH values of whey-mango-based mixed beverages were estimated during 0, 5, 10, 15, 20, and 25 days of the storage period at refrigerated conditions (4 ± 1 °C). During storage, the pH value was significantly (*p* < 0.05) changed based on storage days and formulations. The pH values of formulated whey-mango-based mixed beverages were recorded as sample-1 (4.27 ± 0.07- 3.99 ± 0.08), sample-2 (4.4 ± 0.02–4.21 ± 0.04), and sample-3 (4.58 ± 0.09–4.28 ± 0.03), respectively. There was a significant (*p* < 0.05) decrease in the pH value of the formulations during storage due to a rise in titratable acidity, which is inversely proportional to the pH. The pH value of the prepared whey-mango-based mixed beverages in the current study is in agreement with the findings of Chavan et al. [22]. They developed a whey-based mango beverage and reported a significant decrease in the pH ranging from 4.3 to 4.1 after 30 days of storage at refrigerated temperature. Similarly, Jooyandeh [38] observed a pH decline after 90 days of storage of two naturally carbonated plum (red and yellow) beverages. The decline in pH may be caused by amino and organic acid generation during storage from proteins and lactose. The gradual decline in pH may be regarded as a beneficial characteristic because it possesses antibacterial properties and the low pH inhibits the growth of pathogenic microorganisms [39]. However, beverages remain clear at pH values considerably below the isoelectric threshold (~4.6). Clear beverages containing whey protein are now produced with a pH of less than 4.0 to preserve their clarity [40,41]. Therefore, if the beverage has a pH value between 3.5 and 4.5, a stabilizer such as citric acid might be useful to prevent the whey proteins from aggregating during the heat process [39].

### 3.2. Acidity

The effect of storage and concentration of whey as well as of mango on the acidity of whey-mango-based mixed beverages is shown in Table 2. During the period of storage, there was a progressive increase in acidity. According to the findings, the acidity of the whey-mango-based mixed beverages significantly (*p* < 0.05) increased from 0.41 ± 0.03 to 0.64 ± 0.03% for sample-1, 0.39 ± 0.02 to 0.61 ± 0.04% for sample-2, and 0.27 ± 0.02 to 0.44 ± 0.03% for sample-3 after 25 days of storage at refrigerated condition (4 ± 1 °C). Additionally, the percentage of whey and mango in the formulation showed significant (*p* < 0.05) variations in the acidity of the whey-mango-based mixed beverages. The increase in acidity contents could result from ascorbic acid, which produces organic acids and amino acids through its action on the sugar and protein contents of the beverages. Another possible reason may be the transformation of lactose and proteins into lactic acid, and amino acids resulted in an increase in acidity and a decrease in the pH of beverages [42]. The results are consistent with the findings of Yadav et al. [43], who used acid whey made by acid coagulation of milk and found an inverse relationship between acidity and pH in a whey-based herbal beverage. They concluded that acidity increased from 0.40 to 0.50 after 20 days of storage at refrigerator temperature. Sakhale et al. [44] recently reported an increase in the acidity of ready-to-serve (RTS) mango beverages prepared with acid whey (2% citric acid) after 30 days of storage. The observed values for the acidity of whey-based mixed beverages are also consistent with the findings of Divya [45], who determined that the overall acidity of whey–guava beverages increased with time. Similarly, Saravana and Manimegalai [46] observed a decrease in pH and an increase in acidity after three months of refrigerator storage of a whey-based papaya beverage.

### 3.3. Total Soluble Solids (TSS)

All three samples (sample-1, sample-2, and sample-3) had a considerable increase in the contents of total soluble solids (TSS) across the storage period from 0 to 25 days (Table 2). The maximum increase in TSS (17.15 to 17.50 °Brix) was detected in sample-1, while the lowest increase (18.10 to 18.20 °Brix) was noticed in sample-3. The increase in TSS may be attributed to the conversion of insoluble polysaccharides into reduced sugars. The levels of reducing sugars can also increase due to acid hydrolysis of sugars, which may have resulted in the breakdown of disaccharides into monosaccharides. The hydrolysis of sucrose into inverted sugars may also be an alternative plausible reason for the rapid increase of total soluble solids levels [47]. A similar increasing trend of TSS was observed in a whey-based mango herbal beverage studied by Alane et al. [21], who noticed that the TSS concentration was increased from 15 to 17.2 °Brix after 30 days of storage. The authors suggested that an increase in TSS could be a result of the product’s insoluble part being dissolved in acid (citric and ascorbic) during storage. The increase in TSS was also reported in whey-based mango herbal pudina (*M. arvensis*) beverage [48], whey and apple-based herbal functional ready-to-serve beverage [49], whey-based orange beverage [9], whey-based pineapple and bottle gourd beverage [23], whey-based papaya ready-to-serve beverage [50], and whey-based banana herbal (*Mentha arvensis*) beverage [43]. Similarly, the levels of TSS were also found to increase significantly (*p* < 0.05) in the formulations with the increase in whey and decrease in mango concentrations. However, these results showed inconsistency with previous findings reported by Pandey and Ojha [20].

### 3.4. Reducing Sugars

Reducing sugar contents of the prepared whey-mango-based mixed beverage samples showed significant (*p* < 0.05) effects with regard to formulations and storage periods (Table 2). The reducing sugars levels of prepared beverages ranged from 3.32 ± 0.02–3.67 ± 0.01% (sample-1), 3.14 ± 0.03–3.38 ± 0.03% (sample-2), and 3.01 ± 0.01–3.31 ± 0.01% (sample-3) after 25 days of storage (Table 1). The highest reducing sugars contents were observed in sample-1 (3.67 ± 0.01%) whereas the minimum level was recorded in sample-3 (3.31 ± 0.01%). The progressive increase in the reduced sugar contents of the beverage during the storage period can result from the inversion of non-reducing sugar (i.e., sucrose) to reducing sugar (i.e., fructose) by acid present in the beverage [13]. These findings are in agreement with Pandey and Ojha [20], who prepared mango-based whey beverages and reported similar trending results with the change in the ratio of whey and mango concentrations in the beverage. Similarly, Sakhale et al. [44] developed a whey-based ready-to-serve beverage from mango (Cv. *Kesar*) and confirmed that reducing sugars (%) increased during storage. On the other hand, the present study’s findings contradict previous findings by Barwal et al. [47], who observed a decrease in reduced sugar contents of ready-to-serve beverage drinks from bitter gourd. However, the storage period in that trial was 90 days, which is much longer than the storage time in the current investigation.

### 3.5. Ascorbic Acid (Vitamin C)

Ascorbic acid (vitamin C) is an important nutrient, which has the capacity to antioxidize and defend against free radicals. It is also considered an important indicator of the nutritional quality of the beverage. The ascorbic acid contents of the prepared three formulations varied with the ratio of whey and mango incorporated into the beverages during preparation. With the increasing proportion of whey and decreasing proportion of mango, the ascorbic acid contents decreased significantly (*p* < 0.05) (Table 2). The highest value of ascorbic acid (9.92 ± 0.01 mg/100 mL) was recorded for 60 mL whey and 20 mL in sample-1 whereas the lowest value of ascorbic acid (7.76 ± 0.01 mg/100 mL) for 70 mL whey and 10 mL mango in sample-3. Similar findings were reported by Jakhar [51] in whey and apple-based beverages, Pandey and Ojha [20] in mango-based whey beverages, and Sakhale et al. [44] in whey-based ready-to-serve beverages from mango. However, the ascorbic acid levels of all beverage samples were significantly decreased during storage. This decline could be explained by the fact that ascorbic acid is sensitive to oxygen, heat, and light and oxidizes rapidly in the presence of oxygen. Therefore, the ascorbic acid may have been destroyed during processing and throughout the storage period. Similarly, the development of hydroxymethylfurfural molecules and the degradation of ascorbic acid to carbolic acid, hydroxyl furfural, and dehydroascorbic acid furfural in the beverage is a common phenomenon associated with ascorbic acid degradation during storage [52,53]. Increased moisture contents may also result in ascorbic acid dilution and concentration decline [54].

### 3.6. Solid-Not-Fat (SNF)

The data pertaining to the solid-not-fat (SNF) contents of whey-mango-based mixed beverages affected by formulations and storage period are presented in Table 2. It is clear from Table 2 that the mean SNF content of the whey-mango-based mixed beverage sample was significantly (*p* < 0.05) affected by the addition of whey and mango juice at different levels. The SNF percentage was significantly highest in sample-3, while the lowest SNF contents were recorded in sample-1. The reason may be due to the higher concentration of whey in sample-3 compared to sample-1. In a separate study, Pandey, and Ojha [20] and Sakhale et al. [44] determined the physico-chemical composition of whey and mango-pulp. The authors in both studies expected a higher amount of SNF in whey and mango pulp. Interestingly, they found that whey was better in SNF, while mango pulp did not contain a detectable amount of SNF. Furthermore, the mean SNF percentage was significantly decreased in the present study from 5.78 ± 0.01%–5.20 ± 0.01%, 6.69 ± 0.03%–5.30 ± 0.03%, 8.23 ± 0.01%–5.60 ± 0.02% in case of sample-1, sample-2, and sample-3, respectively after 25 days of storage (Table 2). Our findings support the study of Idan et al. [55], who observed a slight decrease in the SNF contents from 6.479% to 6.468% for grape-flavored whey probiotic beverages after 30 days of cold storage. Sameen et al. [56] reported similar observations that, at the beginning of the storage, the average value of the SNF contents for a carbonated flavored whey drink was 9.54%, which dropped to 9.51% on the 30th day of storage. One reason for a decrease in SNF contents may be the solubilization of the solid proportion under higher acidic conditions [44]. Additionally, the sedimentation of solid parts during the storage period may also contribute to a decrease in SNF levels.

### 3.7. Moisture, Protein, and Fat

The moisture, protein, and fat contents of the prepared beverages (sample-1, sample-2, and sample-3) varied significantly (*p* < 0.05) as storage duration and whey and mango concentration increased (Table 3). The moisture content of formulated beverages ranged from 74.50 ± 0.02 to 87.02 ± 0.03%. There was a noticeable increase in moisture contents with the increased concentration of whey and mango juice, which might be attributed to the addition of different proportions of whey and mango juice to the formulated beverages. The present study findings were comparable with Bhavsagar et al. [57] and David and Kumar [58], who reported moisture content in whey-based beverages from 75.60 to 86.20%. Notwithstanding, the moisture contents increased with storage time. The beverages began to absorb moisture, which was most likely caused by the bottles being opened when samples were required for analysis.

Protein contents of sample-1, sample-2, and sample-3 decreased throughout the storage span from 5.67 ± 0.02 to 5.57 ± 0.02%, 7.36 ± 0.01 to 7.29 ± 0.02%, and 7.66 ± 0.01 to 7.58 ± 0.01%, respectively. An ascorbic acid-induced protein degradation or a change in protein composition to amino acids could explain the decrease in protein contents of whey-mango-based mixed beverages throughout storage time [59]. The addition of whey increases the protein contents of beverages, as evidenced by a linear increase in protein contents with an increase in whey percentage. The highest protein content was reported in sample-3.

Similarly, whey-mango based mixed beverages sample-1, sample-2, and sample-3 had mean fat contents 0.93 ± 0.02%, 1.24 ± 0.05%, and 1.52 ± 0.03%, respectively. A significant (*p* < 0.05) decrease in fat contents from 0.97 ± 0.01 to 0.83 ± 0.01%, 1.30 ± 0.01 to 1.14 ± 0.02%, and 1.57 ± 0.01 to 1.39 ± 0.04% was noticed after 25 days of storage.

Earlier research by Yadav et al. [43] bolstered the current findings, which showed a significant effect of storage on the protein and fat contents of whey-based beverages. Additionally, they explained that the decrease in fat and protein contents of beverages during storage could be related to the synthesis of fatty acids and proteolysis, which diminish the overall solid contents. Yasmin et al. [60] confirmed similar findings, and reported that the protein and fat contents of whey-based fructooligosaccharides-supplemented low-calorie drinks declined dramatically throughout the study. The current findings are also consistent with the study of Jakhar [51]. They developed beverages with different concentrations of whey as well as apple juice and observed that the fat and protein contents reduced as the whey concentration increased. The metabolic interactions and exposure to light and oxygen during the storage of dairy products may result in oxidative damage to the lipids and proteins, lowering their contents in the final products [61].

### 3.8. Carbohydrate and Ash

In the formulated beverages, carbohydrate levels decreased significantly (*p* < 0.05) during 25 days of storage (Table 3). In beverage processing, carbohydrates may be converted to alcohol, organic acids, and/or carbon dioxide by microorganisms such as bacteria, yeast, etc. [62]. Reducing sugars such as glucose and fructose are released during the hydrolysis of saccharides such as sucrose, which may also be a contributing factor for the decrement of carbohydrate contents in the prepared beverages during the storage period [63]. Additionally, with the concentration change in the manufactured beverages, a significant (*p* < 0.05) increase in the carbohydrate contents of sample-1, sample-2, and sample-3 beverages was also found in refrigerated temperature (4 ± 1 °C) storage condition. Bhavsagar et al. [57] also verified the reduction of carbohydrate contents with the increment of whey percentage when manufacturing a pineapple-flavored beverage from chhana whey. Again, the current findings are strongly corroborated in the study conducted by David and Kumar [58], who also reported a reduction in the carbohydrate contents after increasing the whey percentage and decreasing mango pulp concentration during the preparation of the mango-based beverage.

The data pertaining to ash contents of whey-mango-based mixed beverages are shown in Table 3. The mean ash contents in the formulated beverages were found to be 0.81 ± 0.02%, 0.71 ± 0.02%, and 0.61 ± 0.02% for sample-1, sample-2, and sample-3, respectively. The addition of varying amounts of whey and mango juice influenced the prepared beverages’ ash contents. All the samples were found to be significantly (*p* < 0.05) different from each other. This may be due to the incorporation of mango juice, carrot, almond, and green tea extract. The use of fruits and vegetables for the development of whey beverages studied by many researchers i.e., Baber et al. [64], Bhavsagar et al. [57], Hiralal and Raj [65], and Kamte [66] concluded unanimously that the addition of fruits and vegetables for the preparation of whey beverages increased the ash contents. Also, a significant (*p* < 0.05) variation in ash contents was observed after 25 days of storage period. The decrease in ash contents could be due to its use as a metabolic resource for the growth of microorganisms [67]. Mohamed et al. [68] investigated the physical, chemical, and microbiological aspects of papaya functional whey beverage and observed that the ash concentration was 0.65% on day 0 and decreased up to 0.64% on the 30th day of storage.

### 3.9. Minerals

**Figure 1:**
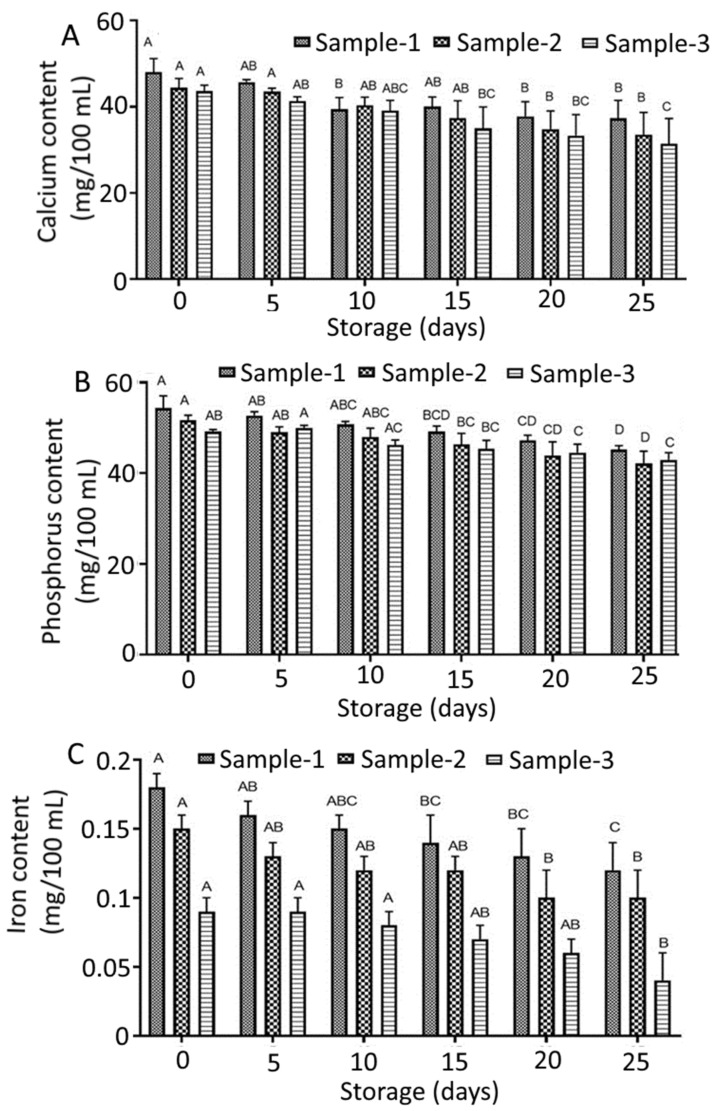
Minerals profile of whey-based mixed beverages: (**A**) calcium content; (**B**) phosphorus content; and (**C**) iron content. All the values are expressed as mean ± standard deviation of three replicates. Different uppercase letters above the same pattern bars indicate significant differences according to Tukey’s multiple comparison test (*p* < 0.05).

Figure 1 illustrates the mineral contents of the prepared beverages during storage for 25 days. The mineral contents of whey-mango-based mixed beverages indicated a substantial effect of formulations, and storage periods on calcium, phosphorus, and iron. The mean values recorded for calcium were 41.37 ± 2.72, 38.97 ± 3.05, and 37.28 ± 3.42 mg/100 mL for sample-1, sample-2, and sample-3, correspondingly after 25 days of the storage period in refrigerated temperature (4 ± 1 °C). Likewise, the observed mean values for phosphorus were 49.86 ± 1.25, 46.80 ± 2.07, and 46.33 ± 1.24 mg/100 mL for sample-1, sample-2, and sample-3, respectively. Besides, the mean values of iron for sample-1, sample-2, and sample-3 were 0.15 ± 0.02, 0.12 ± 0.01, and 0.07 ± 0.01 mg/100 mL, sequentially.

Whey contains a variety of beneficial micronutrients including calcium, phosphorus, and iron, all of which contribute to the enhanced functioning of whey proteins [69]. Whey is an excellent source of bioavailable calcium that improves bone health and reduces the risk of high blood pressure [60,70]. Moreover, calcium derived from whey is easily absorbed in the intestine due to the presence of lactose [71]. In addition to providing bone and teeth strength, phosphorus is extremely effective in performing critical functions for many human organs such as the brain, kidney, heart, and blood vessel walls. Phosphorus works in tandem with calcium to promote bone, gum, and tooth enamel health [72]. Furthermore, iron is also necessary for mammalian nutrition in order to prevent anemia [73]. According to the Bangladesh Institute of Research and Rehabilitation in Diabetes, Endocrine and Metabolic Disorders (BIRDEM) [74], recommended nutrient intake (RNI) for adult males and females of Bangladeshi descent are 1000 mg calcium each, 700 mg phosphorus each, 9 mg and 20 mg iron, respectively per day. Results showed that in the beverage samples, that phosphorus, calcium, and iron contributions to RNI ranged from 12.51–14.29%, 7.55–8.27%, and 1.50–3.30% in adults if 200 mL of the beverage is taken daily.

### 3.10. Sedimentation

In spite of the fact that the immediately processed beverage samples (one day after manufacture) did not exhibit sediments, these were observed in the beverage samples during the second evaluation period (5-day). However, the sedimentation rate was only 1%, which is considered low [2] and remained constant during the storage period (25 days).

### 3.11. Microbiological Analysis

Total plate count (TPC), yeast, and mold count, as well as coliform count, were performed on whey-mango-based mixed beverages at 5-day intervals during refrigerated storage (4 ± 1 °C). The results of the microbiological analysis are summarized in Table 4. Microorganisms respond differentially to the pH conditions that favor their growth. In general, bacteria thrive well in a pH range of 6.25 to 7.25, while yeast and mold grow best in the range of 4.0 to 6.8, depending on their pH requirements [75]. Microbial growth slows as the pH falls below 5, and some bacteria may even die or become damaged [76].

Usually, the TPC test measures the amount of aerobic and mesophilic bacteria in food, drinks as well as beverages [77]. However, the test provides no information regarding the microorganisms that thrived. As a result, the colonies that grow on the agar plate may be distinct from one another. The test is capable of providing data on the raw material, processing, and storage conditions. Hence, it can provide information on product shelf life and safety, contamination, sanitary quality, and organoleptic acceptability [78]. The results related to the TPC of prepared whey-mango-based mixed beverages revealed that the minimum TPC was observed at day 0 for sample-1, sample-2, and sample i.e., 3.32 ± 0.08 (log CFU/mL), 3.33 ± 0.08 (log CFU/mL), and 3.34 ± 0.10 (log CFU/mL), respectively, but reached a maximum TPC at day 25 i.e., 3.45 ± 0.09 (log CFU/mL), 3.47 ± 0.07 (logCFU/mL), and 3.49 ± 0.15 (log CFU/mL), respectively, for sample-1, sample-2, and sample-3. With storage, differences between the TPC of sample-1, sample-2, and sample-3 increased significantly (*p* < 0.05), while samples between themselves did not show any significant differences in the TPC under study on the 5th, 10th, 20th, and 25th day of refrigerated storage. The TPC of the prepared whey-mango based beverage mixed in the current study is consistent with the findings of Gorachiya [79], Sharma et al. [80], Mohamed et al. [68], Yonis et al. [81], and Ismail et al. [82], who reported a similar trend of significant increase (*p* < 0.01) in the TPC of whey-based beverages with an increase of storage period. Whey contains lactose sugar and proteins, which may be conducive to the growth of microorganisms during storage [83].

Similarly, the yeast and mold count of all beverage samples is shown in Table 4. Yeast and mold counts were not detected in all three samples (sample-1, sample-2, and sample-3) at zero-day storage whereas it increased significantly (*p* < 0.05) to 1.71 ± 0.10, 1.78 ± 0.12, and 1.85 ± 0.11 (log CFU/mL) after 25 days of storage. The reduction in the acidic medium may favor the replication of these organisms, resulting in an increase in their number [84]. Similar trending results of yeast and mold count were also observed for whey–guava beverages [81], whey beverages from camel and buffalo milk [79], and *Aloe vera* and coconut water-based whey beverages [85]. On the other hand, coliform count, which is an index of fecal contamination, was undetectable in all samples of whey-mango-based mixed beverages stored at refrigerated temperature conditions (4 ± 1 °C). In this case, the low count of coliform was most likely due to the good hygienic conditions maintained during beverage manufacturing and the absence of fecal contamination. In addition, the severity of heat treatments of whey and the low pH (~4.50) of the whey-mango-based mixed beverages may have also played a role in inhibiting the growth of coliform bacteria [86,87].

According to our understanding, the allowable level of microorganisms in beverages served generally in Bangladesh has not been specified. Therefore, the present study followed the Gulf standard (GS 1016) [88] and National Food Safety Standard for Beverage (GB 7101-2015) [89] reports for the verification of the current findings. According to the GS 1016 and GB 7101-2015 reports, the maximum count acceptable for total plate count is 10^4^ CFU/mL, yeast, and mold 10^3^ CFU/mL, and coliforms 10^2^ CFU/mL for beverages served in the Gulf region and China. It was obvious from the microbiological analysis of the present study that the prepared whey-mango-based mixed beverages had a microbial load in the range that is safe for human consumption and did not surpass the maximum limit set by the Gulf standard (GS 1016) and National Food Safety Standard for Beverage (GB 7101-2015). The prepared beverage samples had a low rate of microbiological growth, which might be attributed to the antibacterial activity of the ingredients included in the formulations. For instance, Gopal et al. [90] reported the antimicrobial activity of green tea extract and identified epigallocatechin gallate (EGCG) as the primary antibacterial component in green tea. Similarly, carrot extracts showed an antimicrobial effect against a range of food-borne micro-organisms, and their antimicrobial activity was not linked to phenolic compounds but was presumably due to a polar components [91]. As there was no significant growth of yeast and mold, and coliform during the storage period, the prepared whey-mango-based mixed beverages can be consumed as normal beverages even after storage for 25 days at the refrigerated condition (4 ± 1 °C).

### 3.12. Sensory Evaluation

Table 5 depicts the sensory scores obtained for all the samples after 25 days. The highest sensory score (6.87) in terms of overall acceptability was obtained by the sample containing whey to mango juice at 60:20 and 65:15 initially, which gradually decreased with the storage period and ended up at 2.83 and 2.81, respectively, on the 25th day of storage. The sample containing the highest amount of whey obtained almost similar scores in color and flavor, except for taste, appearance, and acceptability. The decrease in overall acceptability scores may have been caused by the beverages’ loss of color due to browning, increase in acidity, and change in taste and flavor after storage [92]. However, regardless of the treatment and days of storage, the sensory scores of the samples declined over the storage period. Similar observations have also been reported in the study of Das & Nazni [93], Nedanovska et al. [94], and Yamahata et al. [95].

## 4. Conclusions

From the present study, it can be concluded that whey supplemented with fruits and vegetables could be a potential option for the development of whey-based healthy beverages with optimum sensory characteristics. Results revealed that whey-mango-based mixed beverages met considerable microbiological safety recommendation for consumption for a storage period of up to 15 days under the refrigerated conditions (4 ± 1 °C). In view of the functional properties arising from the bioactive constituents present in fruit and whey, it can be assumed that whey-mango-based mixed beverages could be an interesting product in the constantly growing market of the beverage industry. Therefore, for using whey-mango-based mixed beverages at a commercial scale, further studies should be encouraged on shelf life, improvement of quality through the use of stabilizers and opaque packaging, optimizing the process conditions, and by conducting a clinical trial to ensure safety as well as the beverage’s functional benefits for human health.

## Figures and Tables

**Table 1:** Formulation of whey-mango based mixed beverage.

Ingredients	Sample-1	Sample-2	Sample-3
Whey (mL)	60	65	70
Mango (mL)	20	15	10
Carrot (mL)	10	10	10
Sugar (mL)	6	6	6
Almond (mL)	1.5	1.5	1.5
Green tea extract (mL)	1.5	1.5	1.5
Citric acid (20%) (mL)	1	1	1

**Table 2:** Physicochemical analysis of whey-mango based mixed beverage ^1^.

Parameter	Storage (Days)	Sample-1	Sample-2	Sample-3
Acidity (%)	0	0.41 ± 0.03 ^a A^	0.39 ± 0.02 ^bc A^	0.27 ± 0.02 ^c A^
	5	0.44 ± 0.03 ^a AB^	0.44 ± 0.03 ^bc AB^	0.29 ± 0.02 ^c AB^
	10	0.48 ± 0.03 ^a AB^	0.48 ± 0.03 ^bc BC^	0.32 ± 0.02 ^c AB^
	15	0.51 ± 0.03 ^a BC^	0.52 ± 0.03 ^bc C^	0.36 ± 0.04 ^c BC^
	20	0.58 ± 0.03 ^a CD^	0.55 ± 0.04 ^bc CD^	0.40 ± 0.05 ^c CD^
	25	0.64 ± 0.03 ^a D^	0.61 ± 0.04 ^bc D^	0.44 ± 0.03 ^c D^
pH	0	4.27 ± 0.07 ^a A^	4.44 ± 0.02 ^b A^	4.58 ± 0.09 ^c A^
	5	4.23 ± 0.05 ^a AB^	4.39 ± 0.02 ^b AB^	4.52 ± 0.04 ^c AB^
	10	4.17 ± 0.03 ^a AB^	4.35 ± 0.04 ^bc ABC^	4.43 ± 0.02 ^c BC^
	15	4.13 ± 0.05 ^a B^	4.28 ± 0.03 ^bc BCD^	4.36 ± 0.06 ^c CD^
	20	4.12 ± 0.04 ^a B^	4.26 ± 0.05 ^bc CD^	4.29 ± 0.02 ^c D^
	25	3.99 ± 0.08 ^a C^	4.21 ± 0.04 ^bc D^	4.28 ± 0.03 ^c D^
TSS (°Brix)	0	17.15 ± 0.01 ^a A^	17.50 ± 0.01 ^b A^	18.10 ± 0.01 ^c A^
	5	17.20 ± 0.01 ^a B^	17.60 ± 0.01 ^b B^	18.10 ± 0.02 ^c A^
	10	17.50 ± 0.01 ^a C^	17.80 ± 0.01 ^b C^	18.20 ± 0.01 ^c C^
	15	17.50 ± 0.01 ^a C^	17.80 ± 0.01 ^b C^	18.20 ± 0.01 ^c C^
	20	17.50 ± 0.01 ^a C^	17.80 ± 0.02 ^b C^	18.20 ± 0.01 ^c C^
	25	17.50 ± 0.01 ^a C^	17.80 ± 0.02 ^b C^	18.20 ± 0.01 ^c C^
Reducing Sugars (%)	0	3.32 ± 0.02 ^a A^	3.14 ± 0.03 ^b A^	3.01 ± 0.01 ^c A^
	5	3.45 ± 0.02 ^a B^	3.20 ± 0.01 ^b A^	3.12 ± 0.04 ^c B^
	10	3.52 ± 0.01 ^a C^	3.29 ± 0.02 ^b B^	3.19 ± 0.06 ^c C^
	15	3.59 ± 0.04 ^a D^	3.32 ± 0.02 ^b BC^	3.21 ± 0.02 ^c C^
	20	3.63 ± 0.03 ^a DE^	3.35 ± 0.04 ^b BC^	3.28 ± 0.01 ^c D^
	25	3.67 ± 0.01 ^a E^	3.38 ± 0.03 ^b C^	3.31 ± 0.01 ^c D^
Ascorbic acid (mg/100 mL)	0	9.92 ± 0.01 ^a A^	8.98 ± 0.01 ^b A^	7.76 ± 0.01 ^c A^
	5	8.89 ± 0.01 ^a B^	7.70 ± 0.01 ^b A^	6.73 ± 0.01 ^c B^
	10	8.04 ± 0.01 ^a C^	7.05 ± 0.01 ^b C^	6.38 ± 0.01 ^c C^
	15	7.80 ± 0.01 ^a D^	6.79 ± 0.01 ^b D^	5.95 ± 0.01 ^c D^
	20	7.18 ± 0.01 ^a E^	6.24 ± 0.01 ^b E^	4.89 ± 0.01 ^c E^
	25	6.26 ± 0.01 ^a F^	5.59 ± 0.01 ^b F^	4.01 ± 0.01 ^c F^
Solid-not-fat (SNF) (%)	0	5.78 ± 0.01 ^a A^	6.69 ± 0.03 ^b A^	8.23 ± 0.01 ^c A^
	5	5.75 ± 0.01 ^a A^	6.77 ± 0.02 ^b A^	8.20 ± 0.01 ^c A^
	10	5.67 ± 0.01 ^a B^	6.69 ± 0.02 ^b B^	7.98 ± 0.03 ^c B^
	15	5.59 ± 0.01 ^a C^	6.67 ± 0.02 ^b B^	7.83 ± 0.02 ^c C^
	20	5.48 ± 0.01 ^a D^	6.58 ± 0.02 ^b C^	7.75 ± 0.05 ^c D^
	25	5.20 ± 0.01 ^a E^	5.30 ± 0.03 ^b D^	5.60 ± 0.02 ^c E^

^1^ Results are expressed as mean values ± standard deviation (*n* = 3). Mean values followed by uppercase letters within column and lowercase letters within row indicates significant difference according to Tukey’s multiple comparison test with a probability of 5% (*p* < 0.05).

**Table 3:** Physicochemical analysis of whey-mango based mixed beverage ^1^.

Parameter	Storage (Days)	Sample-1	Sample-2	Sample-3
Moisture (%)	0	74.50 ± 0.02 ^a A^	78.59 ± 0.06 ^b A^	83.09 ± 0.04 ^c A^
	5	75.00 ± 0.01 ^a B^	79.01 ± 0.04 ^b B^	84.02 ± 0.03 ^c B^
	10	75.86 ± 0.03 ^a C^	79.88 ± 0.02 ^b C^	85.47 ± 0.04 ^c C^
	15	78.04 ± 0.01 ^a D^	81.09 ± 0.05 ^b D^	86.66 ± 0.01 ^c D^
	20	78.83 ± 0.02 ^a E^	81.79 ± 0.01 ^b E^	86.91 ± 0.02 ^c E^
	25	79.91 ± 0.02 ^a F^	82.08 ± 0.05 ^b F^	87.02 ± 0.03 ^c F^
Protein (%)	0	5.67 ± 0.02 ^a A^	7.36 ± 0.01 ^b A^	7.66 ± 0.01 ^c A^
	5	5.65 ± 0.01 ^a AB^	7.36 ± 0.01 ^b A^	7.62 ± 0.01 ^c B^
	10	5.65 ± 0.01 ^a AB^	7.33 ± 0.01 ^b AB^	7.62 ± 0.02 ^c B^
	15	5.63 ± 0.02 ^a BC^	7.34 ± 0.01 ^b AB^	7.62 ± 0.01 ^c B^
	20	5.60 ± 0.01 ^a CD^	7.32 ± 0.01 ^b BC^	7.60 ± 0.01 ^c BC^
	25	5.57 ± 0.02 ^a D^	7.29 ± 0.02 ^b C^	7.58 ± 0.01 ^c C^
Fat (%)	0	0.97 ± 0.01 ^a A^	1.30 ± 0.01 ^b A^	1.57 ± 0.01 ^c A^
	5	0.96 ± 0.03 ^a A^	1.29 ± 0.04 ^b A^	1.57 ± 0.03 ^c A^
	10	0.96 ± 0.02 ^a A^	1.27 ± 0.04 ^b A^	1.56 ± 0.02 ^c A^
	15	0.95 ± 0.01 ^a AB^	1.27 ± 0.05 ^b A^	1.53 ± 0.02 ^c AB^
	20	0.89 ± 0.02 ^a BC^	1.19 ± 0.02 ^b B^	1.48 ± 0.03 ^c B^
	25	0.83 ± 0.01 ^a C^	1.14 ± 0.02 ^b B^	1.39 ± 0.04 ^c C^
Carbohydrate (%)	0	18.01 ± 0.02 ^a A^	11.67 ± 0.01 ^b A^	6.98 ± 0.02 ^c A^
	5	17.91 ± 0.02 ^a B^	11.27 ± 0.01 ^b B^	6.47 ± 0.02 ^c B^
	10	17.01 ± 0.06 ^a C^	11.19 ± 0.02 ^b C^	6.09 ± 0.02 ^c C^
	15	14.07 ± 0.02 ^a D^	8.98 ± 0.06 ^b D^	4.07 ± 0.04 ^c D^
	20	12.96 ± 0.02 ^a E^	8.91 ± 0.03 ^b E^	3.98 ± 0.02 ^c E^
	25	12.87 ± 0.01 ^a F^	8.79 ± 0.02 ^b F^	3.45 ± 0.02 ^c F^
Ash (%)	0	0.84 ± 0.01 ^a A^	0.75 ± 0.01 ^b A^	0.67 ± 0.02 ^c A^
	5	0.84 ± 0.01 ^a A^	0.72 ± 0.01 ^b AB^	0.64 ± 0.02 ^c AB^
	10	0.83 ± 0.04 ^a A^	0.71 ± 0.03 ^b AB^	0.60 ± 0.03 ^c BC^
	15	0.79 ± 0.02 ^a AB^	0.71 ± 0.04 ^b AB^	0.60 ± 0.02 ^c BC^
	20	0.79 ± 0.03 ^a AB^	0.70 ± 0.03 ^b AB^	0.59 ± 0.01 ^c BC^
	25	0.77 ± 0.02 ^a B^	0.68 ± 0.01 ^b B^	0.56 ± 0.02 ^c C^

^1^ Results are expressed as mean values ± standard deviation (*n* = 3). Mean values followed by uppercase letters within column and lowercase letters within row indicates significant difference according to Tukey’s multiple comparison test with a probability of 5% (*p* < 0.05).

**Table 4:** Microbiological analysis of whey-mango based mixed beverages during storage ^1^.

Parameter	Storage (Days)	Sample-1	Sample-2	Sample-3
TPC (log CFU/mL)	0	3.32 ± 0.08 ^a A^	3.33 ± 0.08 ^a A^	3.34 ± 0.10 ^a A^
	5	3.34 ± 0.05 ^a AB^	3.35 ± 0.05 ^a AB^	3.37 ± 0.05 ^a AB^
	10	3.37 ± 0.08 ^a B^	3.38 ± 0.09 ^a B^	3.39 ± 0.10 ^a B^
	15	3.40 ± 0.06 ^a C^	3.42 ± 0.07 ^a C^	3.43 ± 0.04 ^b C^
	20	3.43 ± 0.07 ^a CD^	3.45 ± 0.08 ^a CD^	3.46 ± 0.05 ^a C^
	25	3.45 ± 0.09 ^a D^	3.47 ± 0.07 ^a D^	3.49 ± 0.15 ^a D^
Yeast and mold (log CFU/mL)	0	0.48 ± 0.01 ^a A^	0.60 ± 0.02 ^a A^	0.60 ± 0.03 ^a A^
	5	1.00 ± 0.03 ^a AB^	1.15 ± 0.05 ^a AB^	1.20 ± 0.04 ^a AB^
	10	1.26 ± 0.05 ^a AB^	1.36 ± 0.08 ^a B^	1.48 ± 0.09 ^a BC^
	15	1.38 ± 0.06 ^a BC^	1.48 ± 0.08 ^a BC^	1.57 ± 0.09 ^a CD^
	20	1.58 ± 0.08 ^a CD^	1.63 ± 0.06 ^a CD^	1.72 ± 0.11 ^b DE^
	25	1.71 ± 0.10 ^a D^	1.78 ± 0.12 ^a D^	1.85 ± 0.11 ^b E^
Coliform (log CFU/mL)	0	0	0	0
	5	<1 **	<1 **	<1 **
	10	<1 **	<1 **	<1 **
	15	<1 **	<1 **	<1 **
	20	<1 **	<1 **	<1 **
	25	<1 **	<1 **	<1 **

^1^ Results are expressed as mean values ± standard deviation (*n* = 3). Mean values followed by uppercase letters within column and lowercase letters within row indicates significant difference according to Tukey’s multiple comparison test with a probability of 5% (*p* < 0.05). ** below the detection limit of the method.

**Table 5:** Sensory analysis of whey-mango based mixed beverage during storage ^1^.

Sample	Storage (Days)	Color	Flavor	Taste	Appearance	Overall Acceptability
Sample-1	0	8.24 ± 0.03 ^A^	7.28 ± 0.03 ^A^	6.88 ± 0.03 ^A^	5.25 ± 0.03 ^B^	6.87 ± 0.04 ^A^
	5	7.79 ± 0.06 ^B^	7.16 ± 0.04 ^B^	6.39 ± 0.03 ^C^	4.85 ± 0.02 ^D^	6.76 ± 0.17 ^B^
	10	7.42 ± 0.02 ^C^	6.92 ± 0.03 ^D^	6.09 ± 0.03 ^F^	4.21 ± 0.02 ^F^	6.18 ± 0.02 ^D^
	15	6.24 ± 0.01 ^E^	6.18 ± 0.05 ^G^	5.71 ± 0.04 ^H^	4.09 ± 0.03 ^G^	5.53 ± 0.05 ^F^
	20	4.24 ± 0.03 ^F^	3.99 ± 0.02 ^J^	4.09 ± 0.06 ^K^	3.48 ± 0.07 ^L^	3.88 ± 0.10 ^I^
	25	2.89 ± 0.02 ^I^	2.17 ± 0.05 ^I^	3.10 ± 0.03 ^N^	3.09 ± 0.03 ^J^	2.83 ± 0.02 ^K^
Sample-2	0	8.29 ± 0.03 ^A^	7.28 ± 0.04 ^A^	6.71 ± 0.03 ^B^	5.36 ± 0.03 ^A^	6.87 ± 0.06 ^A^
	5	7.83 ± 0.09 ^B^	7.14 ± 0.06 ^B^	6.20 ± 0.02 ^E^	5.09 ± 0.03 ^C^	6.60 ± 0.03 ^C^
	10	7.31 ± 0.08 ^D^	6.85 ± 0.04 ^E^	5.73 ± 0.04 ^H^	4.88 ± 0.02 ^D^	6.20 ± 0.03 ^D^
	15	6.22 ± 0.04 ^E^	6.18 ± 0.05 ^G^	5.20 ± 0.08 ^I^	4.26 ± 0.03 ^F^	5.41 ± 0.07 ^G^
	20	4.13 ± 0.06 ^G^	3.78 ± 0.09 ^K^	3.91 ± 0.06 ^L^	3.69 ± 0.02 ^H^	3.86 ± 0.06 ^I^
	25	2.99 ± 0.02 ^H^	2.11 ± 0.05 ^I^	3.07 ± 0.04 ^N^	3.02 ± 0.05 ^K^	2.81 ± 0.02 ^K^
Sample-3	0	8.23 ± 0.05 ^A^	7.25 ± 0.04 ^A^	6.31 ± 0.02 ^D^	5.21 ± 0.02 ^B^	6.73 ± 0.04 ^B^
	5	7.79 ± 0.02 ^B^	6.99 ± 0.02 ^C^	6.24 ± 0.03 ^E^	5.13 ± 0.07 ^C^	6.55 ± 0.04 ^C^
	10	7.33 ± 0.02 ^D^	6.45 ± 0.01 ^F^	5.88 ± 0.01 ^G^	4.58 ± 0.03 ^E^	6.05 ± 0.03 ^E^
	15	6.20 ± 0.02 ^E^	5.77 ± 0.02 ^H^	4.99 ± 0.02 ^J^	4.08 ± 0.03 ^G^	5.25 ± 0.03 ^H^
	20	4.30 ± 0.01 ^F^	4.12 ± 0.01 ^I^	3.26 ± 0.03 ^M^	3.13 ± 0.02 ^J^	3.67 ± 0.05 ^J^
	25	2.83 ± 0.02 ^I^	2.18 ± 0.03 ^L^	3.10 ± 0.03 ^N^	2.85 ± 0.02 ^L^	2.75 ± 0.03 ^K^

^1^ Results are expressed as mean values ± standard error (*n* = 30). Mean values followed by uppercase letters within column indicates significant difference according to Tukey’s multiple comparison test with a probability of 5% (*p* < 0.05).

## Data Availability

Data are available within the article.
